# The function and structure of the cerebrospinal fluid outflow system

**DOI:** 10.1186/1743-8454-7-9

**Published:** 2010-06-21

**Authors:** Michael Pollay

**Affiliations:** 1 14205 W Via Tercero, Sun City West, AZ 85375, USA

## Abstract

This review traces the development of our understanding of the anatomy and physiological properties of the two systems responsible for the drainage of cerebrospinal fluid (CSF) into the systemic circulation. The roles of the cranial and spinal arachnoid villi (AV) and the lymphatic outflow systems are evaluated as to the dominance of one over the other in various species and degree of animal maturation. The functional capabilities of the total CSF drainage system are presented, with evidence that the duality of the system is supported by the changes in fluid outflow dynamics in human and sub-human primates in hydrocephalus. The review also reconciles the relative importance and alterations of each of the outflow systems in a variety of clinical pathological conditions.

## Review

### Introduction

The functional anatomy of the structures responsible for the return of cerebrospinal fluid (CSF) to the general circulation is based on somewhat conflicting evidence as to their location, anatomical features, and functional capabilities. The dual outflow systems for the egress of CSF from the intracranial CSF compartment control the balance between CSF production and drainage that ultimately impacts the constancy of the cerebral environment. This basic information has clinical implications as to the understanding of the basis and clinical consequences of derangements of the CSF outflow systems. Our present understanding of the two major functional CSF drainage systems evolved separately with recognition of the AV route occurring well in advance of our understanding of the importance of the lymphatic outflow pathway.

### Early history of the study of cranial and spinal arachnoid villi

Both Vesalius and Willis appreciated the presence of the arachnoid granulations in the 16^th ^and 17^th ^century. The detailed dissection of Pacchioni in 1705 revealed for the first time their relationship to the sagittal sinus, which suggested to him a secretory role for these structures. Luschka in the later part of the 19^th ^century again noted the arachnoid structures penetrating the lacuna of the sagittal sinus, which suggested a special function for these arachnoidal projections. He also appreciated that the arachnoid granulations represented enlargement of the normal villi of the arachnoid [1]. Trolard confirmed his findings in 1870 that described the projections into both the lacuna lateralis or directly into the superior sagittal sinus [2]. At almost the same time Quincke followed the distribution of cinnabar injected into CSF of animals and observed, with the microscope, the material enmeshed within the arachnoid granulations, which suggested to him the role of the villi in the removal of the CSF [3]. Key and Retzius [4] later confirmed this conclusion in humans following the injection of colored gelatin in the brain specimens and finding that it enters the villous structures and enters the lacuna lateralis and the venous sinus so as to return the CSF back to the blood. It is also interesting to note that some of the colored tracers (trypan blue) in his experiments were found in the cervical lymph nodes.

Cushing (1901) initially believed that the arachnoidal projections had to be a valved structure in order to meet the requirements for the one-way transfer of CSF into the systemic circulation. He was later convinced by Weed (1914) that these structures represented a semi-permeable blind diverticulum interspersed between the venous blood in the cerebral sinuses and the CSF in the subarachnoid space [5]. Presumably both the osmotic and hydrostatic differences operated to filter the fluid from the subarachnoid space into the venous sinuses. In an early review, however, Davson [6] concluded that the physiological studies demonstrating removal of protein and particulate matter from the subarachnoid space into the cranial sinuses made the notion that the arachnoid villus (AV) was a blind semi-permeable pouch untenable and that the only force required to provide unidirectional passage of fluid into the venous blood was a favorable differential hydrostatic pressure. It was almost a half century later that Weed's view concerning the form and function of the AV was overturned and a new concept concerning the functional anatomy of CSF drainage from the cranial subarachnoid space was proposed [5].

It was well into the 20th century before the functional relationship between the spinal AV and the dorsal root veins and the lymphatic system was fully appreciated although Elman (1923) described the subarachnoid space around the dorsal root of the spinal nerves that contained clusters of arachnoidal cells [7]. He also observed the distribution of Prussian blue granules, following subarachnoid injection, in these clusters, regional spinal dura and in the regional spinal veins. Wisclocki [8] using chemical tracers described a rich plexus of vessels around the dorsal spinal root that he found similar to the intracranial venous sinuses in form and in proximity to the spinal subarachnoid space. This suggested a meningeal relationship with these venous channels similar to that found in the cranial compartment. Greater appreciation of this relationship and potential function in CSF drainage was to occur somewhat later in the mid twentieth century.

### A newer concept of the arachnoid villi drainage system

**Figure 1:**
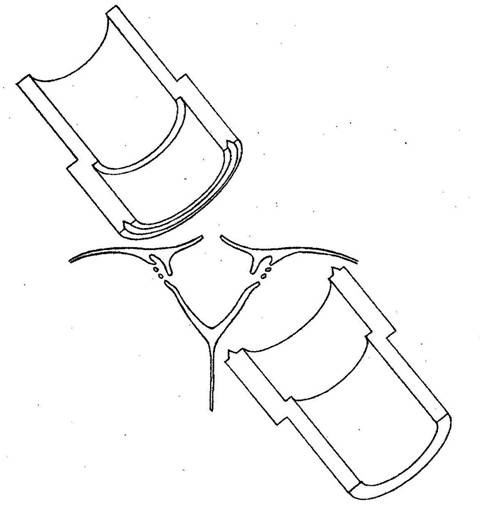
**Experimental arrangement of chamber enclosing arachnoid villi**. Upper chamber enclosing sinus side of dura containing arachnoid villi while lower chamber the external surface of the sinus dura (reproduced with permission from Welch and Friedman [5]).

**Figure 2:**
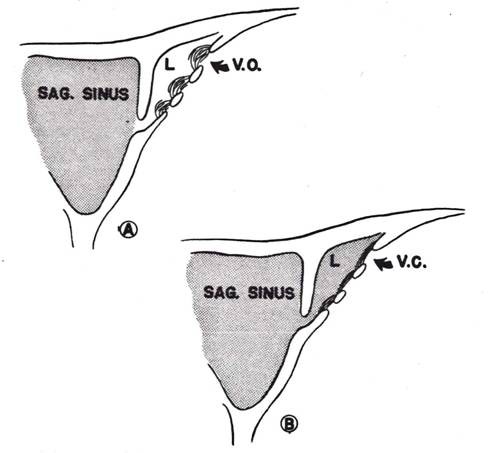
**Cerebrospinal fluid valves after reversal of CSF-to-blood pressure gradient**. A: Open villus (VO) structure when pressure gradient is positive. B: collapsed villus (VC) when the gradient is negative (reproduced with permission from Welch and Friedman [5]).

**Figure 3:**
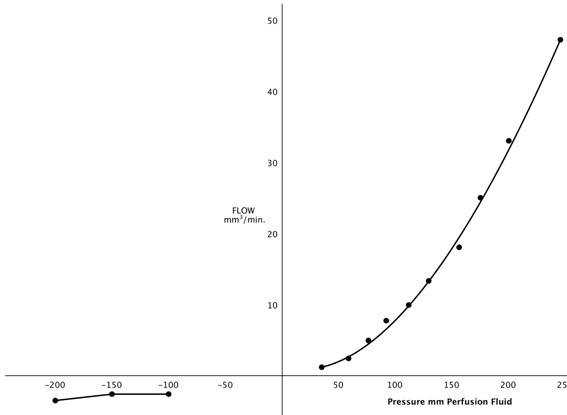
**Flow-pressure curve through dural disc containing arachnoid villi**. Right side: normal flow direction-subarachnoid space to lumen of sagittal sinus. Left side: Flow from sinus to subarachnoid space (reproduced with permission from Welch and Friedman [5]).

The revision of the old view of the cranial CSF outflow system began with the studies of Welch and colleagues on the isolated AV and the concept of CSF valves. It appeared to Welch and Friedman [5] that Cushing's first notion concerning the AV was, in fact, correct. This was based on the perfusion of the isolated cranial AV in the green monkey (Figure 1). They demonstrated from light microscopic studies that the villus consisted of a labyrinth of open tubes when the tissue was fixed at normal physiological pressures. These arachnoidal tubes were effaced when flow of the fixation fluid was in the reverse direction (Figure 2). The physiological data in this study supported this anatomic view of the CSF system with flow in the direction of the sinus beginning at 20 to 50 mm H_2_0 with a curve convex to the pressure axis (Figure 3). The flow of perfusion fluid in response to pressure did not occur in the reverse direction as might be expected from the anatomical arrangement presented. Welch and Pollay [9] later demonstrated the flow of particles through the AV using the same experimental arrangement. These studies demonstrated that particles in micrometer dimensions were capable of traversing the sinus interface. In addition, raising the protein concentration of the perfusate did not negate flow from the meningeal to the sinus side of the preparation although the increased viscosity of the fluid did modestly slow the rate of flow as expected. This finding supported the earlier studies, which showed the AV interface to be permeable to protein and particulate matter. It was also observed that the addition of a surface-activating substance (Tween 80) significantly lowered the opening pressure required to initiate flow from meningeal to sinus side of the experimental preparation. This observation suggested the opening pressure of the tubular passage is due to the inherent stickiness of the valvular structure. The original studies in the green monkey were later extended to a canine model with similar results [10].

### Developmental anatomy of the arachnoid villi

It is generally believed that the structures derived from arachnoidal tissue (arachnoid villi, AV) are initially a microscopic-sized projection that does not fully penetrate the dural wall of the cranial venous sinus. With maturation they continue to grow in size and penetrate the dura mater with changes in the functional morphology of these structures, which then may be seen without magnification. They are properly called arachnoid granulations or Pacchionian bodies at this stage. It has been proposed that maturity and increasing the pressure within the CSF system provides the stimulus for this transformation [11]. In man these structures are present in large numbers along the cerebral venous sinuses but especially the superior sagittal sinus in the region where the parietal occipital vein enters the venous sinus cavity. They are also seen with some regularity and density along the transverse venous sinus especially in the region of the confluence of the sinuses. Arachnoid villi in the region of the *sella tursica *have also been observed in humans with a well-developed intercavernous sinus or venous plexus [12].

In human fetuses and newborn, Gomez *et al *[13] demonstrated in the 26-week fetus, dural depressions in the venous dural wall. These depressions contained arachnoidal cell clusters penetrating between the dural fibers with some reaching a sub endothelial position. By 35-weeks these depressions were converted into simple protrusions through the dura with characteristics of AV. From 39-weeks onward, the number and complexity of the villi increased. These findings are generally in agreement with less extensive earlier studies concerning the appearance of the dural depressions and fit the observed maturation of the subarachnoid space by the 30th week of fetal life [3]. It is the relative paucity of these structures in the late fetal and early postnatal period that suggests the importance of the lymphatic system in CSF drainage during this early period of development [13,14].

In senescence, the AVs occlude and degenerate and the arachnoid membrane thickens. It occurs along with degenerative changes in the choroid plexus. These changes in the CSF system result in stagnation of the CSF and possibly play an important role in dementia of the aged [15,16].

### Morphological features of cranial arachnoid villi

**Figure 4:**
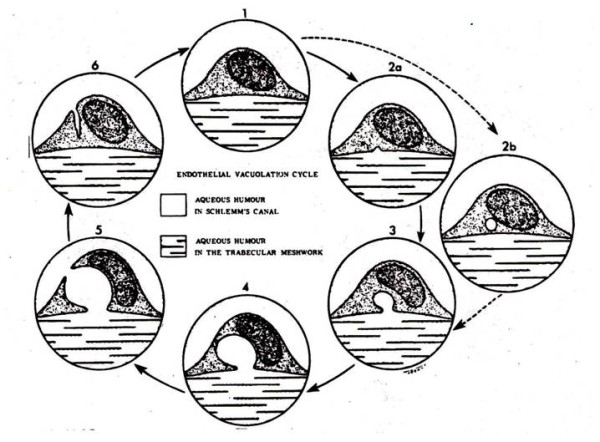
**Diagrammatic representation of intracellular vacuolation process of cells in canal of Schlemm (reproduced with permission from Tripathi **[20]**)**.

**Figure 5:**
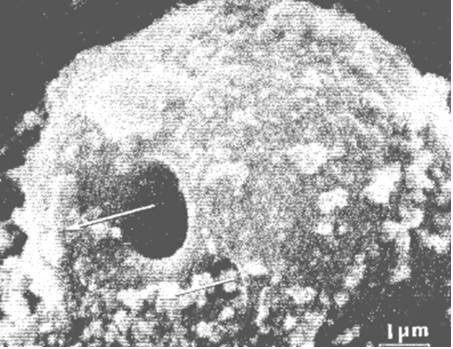
**Scanning electron micrograph of giant vacuole in mesothelial cell lining of the arachnoid villus seen from the apical aspect with passage of tracer material (colloidal suspended Thorotrast), seen here through the natural opening on the apical surface of the vacuole (arrows; reproduced with permission from Tripathi **[19]**)**.

**Figure 6:**
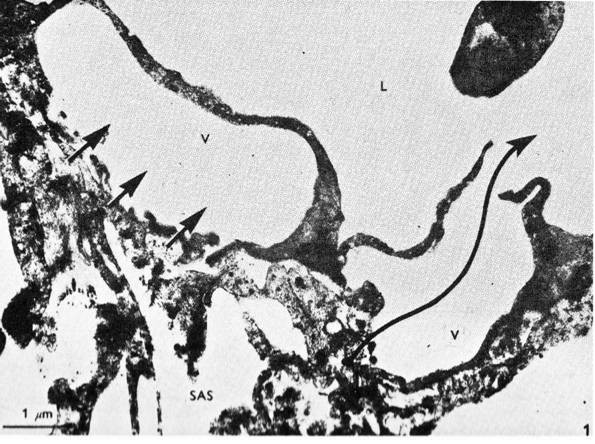
**Electron micrograph of the mesothelial cells lining the arachnoid villus showing empty giant vacuole (V)**. The vacuole on the right has both basal and apical openings, thus constituting a vacuolar transcellular channel (long arrow). The vacuole on the left has basal opening only (short arrows). SAS: subarachnoid space; L: dura lacuna (reproduced with permission from Tripathi [19]).

The correlation between the new concept of an open pathway between CSF and venous sinus blood and the morphological construct presented by Welch and colleagues, using light microscopic methods, were not universally accepted [5,10]. This new concept of open pathway CSF drainage was evaluated, using both transmission electron microscopy (TEM) and scanning electron microscopy (SEM) in a series of papers in both primates and lower animals. These studies proposed conflicting views of the nature of the junction between venous blood and CSF. The earlier studies in using both light and enhanced microscopy appeared to support the view that there was no discontinuity of the sinus endothelium and therefore any movement of CSF into the blood via the AV would necessarily be through the intact endothelial cells although repeat studies showed some interendothelial clefts which allowed penetration of horseradish peroxidase [3,17]. In sheep and dogs, Gomez *et al*, using increasing fluid pressures in the sinus and CSF, observed endothelial intercellular clefts [18]. Pinocytosis was also observed and was similarly pressure dependent. Tripathi [19] demonstrated that when the tissue was fixed at a low or negative differential pressure between the subarachnoid space and the cranial venous sinus there was continuity of the endothelial surface over the arachnoidal interface with many microvilli. When tissue fixation occurred under normal and increased pressure, large intracellular vacuoles were observed, which had an evolving open connection between the CSF and venous blood. Tripathi (1968) first described this concept for endothelial cell vacuolation in the canal of Schlemm but then applied it to the AV outflow system (figure 4) [3,20]. The opening of the vacuoles as a large pore on the surface of the endothelial cells is demonstrated in figure 5. In figure 6, the electron micrographs of the AV demonstrate the connection of these vacuoles with the subarachnoid space and the dural lacuna. These histological observations were later confirmed by Levine *et al *[21]. The intracellular vacuoles were believed to be the major pathway for the bulk drainage of CSF and particulate matter. The arachnoidal pores, however, were not seen on reversal of the pressure gradient used in tissue fixation. Based on their own histological studies in human *post mortem *material and their review of the literature, Upton and Weller [22] believed that the anatomy of the AV of animals and primates are somewhat different and caution should be used in extrapolating between species. They especially noted the significant role of pinocytosis in lower animals as compared to the role of transcellular vacuoles in monkey and man. In general, the literature has supported the notion that drainage of CSF via the arachnoid granulations is by endothelial pinocytosis and vacuolization as well as extracellular cisterns [23]. In the most recent studies, using an *in vitro *or *ex vivo *arachnoid granulation membrane model, derived from human *post mortem *specimens, Grzybowski *et al *[24] and Glimcher *et al *[25] demonstrated the morphological basis for CSF pathways in these biologically viable transplants that conform to the earlier studies mentioned above.

### Dynamic studies of CSF outflow via the arachnoid villi

As mentioned earlier, Welch and colleagues demonstrated in an isolated arachnoid granulation perfusion system, unidirectional flow from meninges to sinus and virtually none in the reverse direction (Figures 2 and 3). It was also found that particles up to 7.5 μm in size passed through the CSF-blood interface [9]. In a series of elegant studies utilizing the harvested AV and arachnoid membrane, Grzybowski and associates [24,25] measured the cellular hydraulic conductivity under normal physiological pressures (Table 1). The experiments demonstrated that in both the *in vitro *and in a later study using an *ex vivo *tissue preparation there was directional flow similar to that found in the experimental model of Welch and Friedman [5,24]. They also found their occluding membrane specimens to be biologically viable and stable during the perfusion study. In addition, utilizing the *ex vivo *model, particle movement (0.1 to 2.0 μm) was shown to cross the membrane in the physiological direction. The establishment of the legitimacy of this experimental methodology should allow a more complete understanding of the movement of a variety of biologically important substances from CSF into the systemic circulation.

**Table 1:** Summary for arachnoid granulations perfused *in vitro *in the physiological (B to A direction) and non-physiological (A to B direction)

Direction of Perfusion	Pressure (mm Hg)	Flow Rate (μl/min)	**Average cellular L**_**p **_**μl/min per mmHg/cm**^**2**^
Average A→B	3.33 ± 0.29	0.52 ± 0.29	0.28 ± 0.16

Average B→A	3.15 ± 0.08	4.3 ± 0.53	4.50 ± 0.53

p-value	0.35	7.40E-08	2.09E-07

Data are average cellular hydraulic conductivity (L_p_). Modified and reproduced with permission from Grzybowski *et al *[24].

The pressure differential required to drain CSF from the subarachnoid space into the superior sagittal sinus via the AV has been shown to vary between 3 and 5 mm Hg. In human subjects, using infusion of artificial CSF, the pressure in the sagittal sinus (SSVP) remains constant over a wide range of artificially raised CSF pressures (CSFP) [3]. The range was well within that seen clinically. Shulman *et al *in a canine model showed that under normal conditions the mean CSFP, SSVP and torcular venous pressure (TVP) to be 147, 90.3, and 46 mm H_2_O respectively [26]. The SSVP is therefore about 60% of the CSFP in the normal animal. In the hydrocephalic dog the ratio of CSFP to SSSP is 0.98 while in the normal animal it is 0.61. Under these conditions, the TVP/CSFP ratio changes little, which is probably due to the protection afforded by the encasement of the torcula in bone in the dog. There have been numerous studies relating CSF pressure and CSF absorption in man and animals under normal conditions and in hydrocephalus. Both the studies of Mann *et al *in rat and McComb *at al *in rabbit, using radiolabeled inulin and serum albumin respectively, have demonstrated the lack of CSF movement over the cerebral hemispheres or into the superior sagittal sinus at physiological CSF pressures [27,28]. It should be appreciated that in the animals used in these studies, the AV were usually absent or scanty at the superior sagittal sinus sites but were observed mostly at the skull base. These findings will be discussed in relation to the olfactory nerve drainage system after presenting the anatomy and physiology of the lymphatic outflow system.

### Functional consequences of morphological changes in arachnoid villi

Although it has been shown that hydrocephalus may follow infection of the meninges, subarachnoid hemorrhage and infusion of FGF-2 (fibroblast growth factor) into the CSF, it is not clear whether the alterations in the CSF circulation and drainage are due to pathological changes in the subarachnoid pathways or to specific changes in the dual outflow system (AV and lymphatic). In *post mortem *studies on the response of the AV to subarachnoid hemorrhage it was found that the observed proliferation of the arachnoidal cells triggered by an inflammatory reaction or blood-clotting products may result in obstruction of CSF flow via the AV into the cranial venous sinuses [29]. It is not possible to rule out the effect on the subarachnoid pathways or drainage into the olfactory lymphatic system following subarachnoid hemorrhage. This difficulty is also noted in evaluating the specific role of the pathological changes in the subarachnoid space, AV and the lymphatics in the development of hydrocephalus following a meningeal infection. The functional consequences of total, or almost total, absence of the AV in humans have been reported to result in the development of hydrocephalus [30]. In these proven cases, the development of the pathways utilized by the lymphatic system and spinal AVs was not evaluated and therefore these may also have played a role. The same problem exists when CSF absorption is affected by a marked increase in the viscosity of the circulating fluid (e.g. high protein content). That is, the altered absorption could be at either the AV or the subarachnoid and perineural lymphatic pathways.

There are also morphological and functional changes in the AV that effect CSF absorption leading to variable changes in intracranial pressure (ICP). In studies in immature animals it has been noted that vitamin A deficiency can lead to the thickening and infiltration of the dura mater around the AV with mucopolysaccharides. This appears to be associated with diminished CSF absorption and increased ICP secondary to the development of hydrocephalus [31]. The adverse effect of hypovitaminosis A has been shown in ventriculo-cisternal perfusion studies in the bovine model to be due directly to diminished absorption by the AV [32]. It has been reported in infants, that secondary hypovitaminosis can lead to an increase in ICP and a bulging fontanel [33]. Toxic levels of Vitamin A can also affect the morphology and function of the AV, choroid plexus and ependymal epithelia. The AV was reduced in size in the affected animals compared to controls and the arachnoidal cap was thinner and smaller. It was suggested that these changes were associated with an increase in CSF outflow with alteration in the functional morphology of the choroid plexus leading to low ICP [34]. Generally, toxic levels of retinol in the CSF may result in an idiopathic intracranial hypertension syndrome [35].

### The spinal arachnoid villi

**Figure 7:**
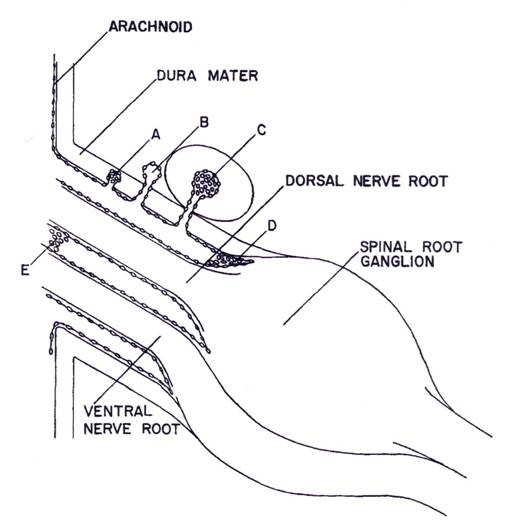
**Diagrammatic representations of the types of meningeal and vascular relationships found in spinal nerve roots**. A: arachnoidal cells within dura mater. B: complete penetration of arachnoid villus into interstitium surrounding spinal root. C: penetration of arachnoid villus into epidural spinal vein D and E arachnoidal proliferations within subarachnoid space (reproduced with permission from Welch and Pollay [37]).

The early studies of the spinal AV demonstrated the presence of the arachnoidal clusters and projections into and through the dura mater in the region of the dorsal root ganglia but they did not fully relate this to the venous vessels in the region or the possibility that these structures represented a site of CSF outflow [3]. Elman appreciated that these structures were similar to the cranial villi and noted the relationship with regional venous structures [7]. Wislocki considered these spinal venous sinuses to be the equivalent to those of the cranial venous sinuses and the special relationship to the arachnoidal villi [8]. Brierley and Fields, using an India ink tracer injected into the CSF demonstrated that the tracer reached the spinal epidural space and the regional lymphatics [36]. In 1961, Welch and Pollay re-visited, in the green monkey, the anatomical relationship of the dorsal root AV and the regional veins [37]. In figure 7, the variety of this relationship is shown. It was apparent from this study on the green monkey that the AV more commonly penetrated the epidural space but was also observed penetrating the thin walled veins found around the dorsal root ganglia. These findings and the earlier studies utilizing tracer movement from the spinal subarachnoid space into the adjacent vascular structures support the anatomical basis of the spinal AV outflow system. The significance of the lymphatics in cranial and spinal outflow was not fully appreciated at that time. In the monkey, the spinal AVs found in relation to venous structures were observed in only 16% of the roots studied. Gomez *et al *reported a somewhat higher incidence of spinal AV in dog and sheep [38]. Kido *et al *found in human cadavers that the concentration of spinal AV was more common in the thoracic region [39]. Tubbs *et al *revisited the relationship between spinal AV and adjacent venous structures in adult human cadavers [40]. They did not find AV on every root but the greatest concentration was found in the lumbar region and all in close relationship to the regional venous vessels. There was also a direct relationship between the size of the adjacent radicular vein and the presence and number of the AV.

Marmarou *et al *[41] working in cats, separated the cranial from the spinal compartment with an inflatable balloon and found that the spinal compartment accounted for 16% of CSF absorption, also that the spinal absorption appeared similar to cranial absorption in that no differences were discernable on the basis of pressure dynamics. In sheep, it was shown that the ratio of cranial to spinal clearance of radiolabeled iodine into the CSF compartment varied with the method used in the study, bolus injection or cranial and spinal infusion [42]. Expressed as a percentage of total CSF drainage the ratio of cranial to spinal clearance by perfusion, bolus injection and introduction by reservoir into both cranial and spinal compartments was estimated to be 75:25, 88:12, and 75:25, respectively. It was concluded that ca. 25% of CSF drainage is via the spinal AV. In human subjects, using lumbar puncture and radionuclide cisternograpy, Edsbagge *et al *[43] calculated CSF formation and drainage with the measurement of spinal radionuclide activity in young healthy humans. The rate of tracer activity decline was about 20% in the first hour and enhanced by physical activity in the human test subjects. The mean CSF production was ca. 0.35 ml/min. Based on nuclide activity reduction in the spinal compartment, the spinal absorption was 0.11 to 0.23 ml/min, based on a net inflow of ventricular CSF into the subarachnoid space of 0.45 and 0.48 ml/min [43]. These values were somewhat higher than previously reported. The unsettled issue was whether the drainage via the AV was into the lymphatics that are found around the spinal roots or into the veins in which some AV penetrate. Most tracer studies support the lymphatic connection being of greater importance in this setting [36,42].

### The early history of the study of lymphatic drainage of cerebrospinal fluid

Although Schwab (1869) observed that substances injected into the subarachnoid space could, at a later time, be found in the cervical lymph nodes, it was Key and Retzius who demonstrated the pathway the tracer used to reach the cervical lymphatic system [4].

**Figure 8:**
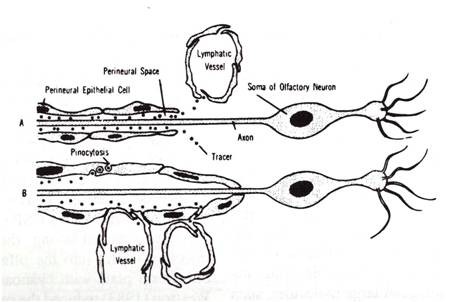
**Diagrammatic representations of two models of olfactory perineural pathway to nasal lymphatic outflow system (reproduced with permission from Jackson *et al ***[48]**)**.

**Figure 9:**
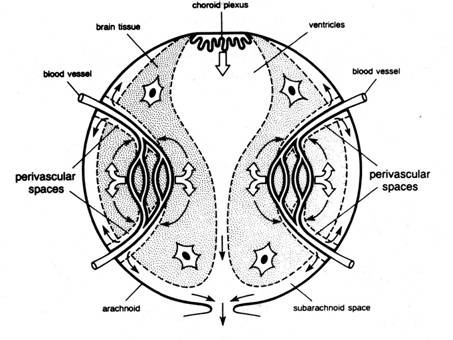
**Schematic diagram illustrating a model of interstitial fluid turnover in the brain based on secretion of cerebral ISF by the blood brain barrier (open arrows) and bulk flow of ISF from brain to CSF via perivascular spaces (curved arrows)**. CSF is secreted by the choroid plexus (open arrows) and drains with ISF from the subarachnoid space into venous blood and lymph (reproduced with permission from Cserr *et al *[51]).

**Figure 10:**
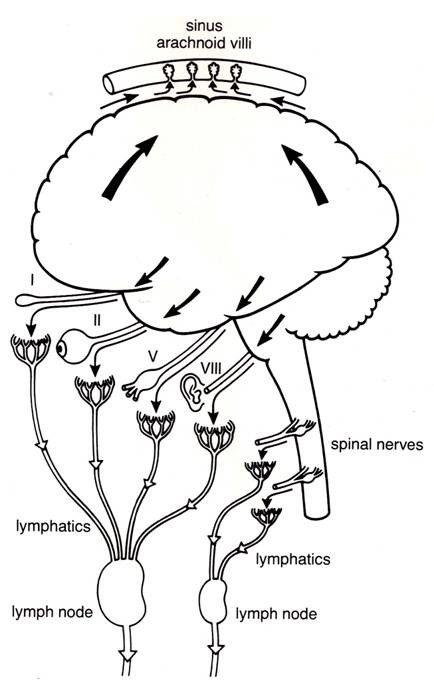
**Schematic of perineural pathways along cranial nerves for subarachnoid CSF- lymphatic connections (thin curved arrows) and into cranial venous blood via arachnoid villi (large curved arrows; reproduced with permission from Cserr *et al ***[51]**)**.

**Figure 11:**
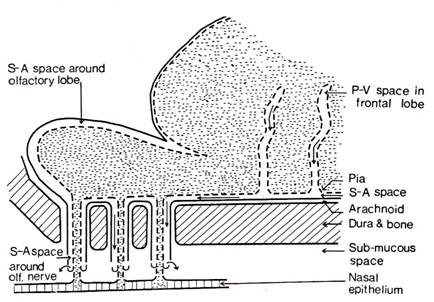
**Schematic of CSF outflow along subarachnoid pathways into nasal lymphatics via olfactory nerve perineural pathway after injection into caudate nucleus (reproduced with permission from Bradbury *et al ***[57]**)**.

Over the next 30 years numerous authors demonstrated in live animals, utilizing a variety of colored or radiolabeled tracers, that there was a route along the olfactory and optic nerves, which eventually led to accumulation of tracers in the cervical lymph nodes [3,44]. Utilizing x-ray visualization, the egress of thorotrast and brominal was shown first by Mortensen and Sullivan (1933) and then Faber (1937), in the living dog and rabbit respectively, that the passage was by way of the olfactory nerves into the cervical lymphatic system usually within a five-hour period following subarachnoid injection [45,46]. Au^198^, injected into the sub-mucosal tissue of the rabbit, was found to penetrate through the cribiform plate into the basal cisterns and frontal lobe [47]. Jackson *et al *[48] presented an anatomical schematic of an open and closed perineural cuff model for drainage of CSF into the cervical lymphatic system via the olfactory perineural space (figure 8). At the same time, it was shown experimentally in rabbit and cat, that the lymphatic system is able to remove both protein and red cells from the CSF [49,50]. Some 25 years later, a series of studies both in cat and rabbit confirmed the early research as to the importance of the lymphatics in the drainage of CSF. Cserr *et al *observed the importance of the lymphatic pathway in rabbit, sheep and cat using a radio-labeled albumin tracer injected into the CSF or directly in the brain [51]. They found that between 14-47% of the injected tracer into brain passes through the lymphatic system. They also suggested that the lymphatic CSF outflow system could represent the afferent arm of the immune response to antigens. These authors implied that there is a continuous and highly regulated communication between brain and the immune system [52]. In a rat model, Weller *et al *also demonstrated the importance of the lymphatic drainage system in the drainage of fluid from the interstitial space of the cortex [53]. It appears that the drainage of cerebral interstitial fluid into the CSF is via the perivasular channels and subsequently entering the lymphatic system through the cribiform plate (figure 9). This supports the view that this pathway serves a primary role in the turnover of the cerebral interstitial fluid. There were also a number of studies, which propose potential drainage sites along many of the other cranial nerves [51,52,54-56]. These are shown schematically in figure 10. Bradbury and Cole observed a significant concentration of I^125 ^-albumin in the deep cervical lymph nodes following a single lateral ventricle injection, which was quite substantial in both the rabbit (14.4%) and cat (12.9%) [56]. The passage of tracer into the orbit also occurred but was quantitatively small and was not found in the cervical lymphatics at 6 hours if at all. In a later paper, Bradbury *et al *[57] presented a schematic of the hypothetical route for CSF drainage of a protein tracer injected into brain, which then entered the subarachnoid CSF by way of the perivasular spaces (figure 11). It appears from these earlier studies that the drainage of the interstitial fluid of the brain is primarily if not exclusively via the nasal lymphatic outflow system and of importance in not only modulating brain fluid volume, but also providing a highly regulated connection between the brain and the immune system [51,52,57]. The turnover of the cerebral interstitial fluid represents about 10% of the total turnover of the CSF compartment [3]. The rate of transfer of CSF from the CSF compartment to the lymphatic outflow system is also adversely affected by a head up position of the animal, obstruction of the cribiform area, and by higher molecular weight tracers [58].

Almost a decade would past before more quantitative evidence became available to support the importance of the lymphatic CSF outflow system in maintaining the cerebral environment and its relative importance in global CSF outflow in comparison with the role of the AV [44].

### Functional morphology of the CSF lymphatic drainage system

#### Anatomical considerations

The early qualitative studies suggested that the anatomical basis for the passage of tracers consisted of the perineural spaces of certain cranial nerves, which on exit from the skull allowed the movement of CSF, originating from the basal subarachnoid space, to enter either the interstitial tissue or directly into the cervical lymphatic system. This view was generally limited to the CSF drainage into the nasal lymphatics via the perineural sheath of the olfactory nerve after penetrating the cribiform plate although incomplete information was found for the ocular or auditory cranial nerves [54-56]. Ultrastructual studies of the olfactory nerve of the rabbit, following the infusion of horseradish peroxidase (HRP), revealed that HRP moved along the perineural space and flowed into the lymphatic vessels, mucous glands, intercellular spaces of the nasal epithelium and venules [59]. In *post mortem *examination in humans following subarachnoid hemorrhage these perineural spaces contained red cells both distally and proximally in the olfactory nerves. Iron pigments were also found in the cervical lymph nodes [60]. Kida *et al *found carbon particles along the subarachnoid space into the nasal cavity, orbit, and cochlea but only the nasal route showed direct communication of cranial CSF with lymphatics [61]. Using HRP as a marker, a connection has been demonstrated between the subarachnoid spaces and the *scala vestibuli *lymphatics [55]. The anatomy of the pathway along the optic nerve is less clear but using contrast material infusion it has been shown that there is leakage at the distal end of the optic nerve sheath (ONS). At the terminal (distal) end of this sheath the neuroendothelial cell layer is quite thin and there are a number of intercellular filaments and pore-like openings [62]. Following injection of HRP into spinal theca, Gomez *et al*, observed HRP in the subarachnoid space around the optic nerve where it penetrated within the nerve, occupying extracellular spaces between the nerve elements, up to the area of the lamina cribosa. After crossing the perineural sclera it spread into the choroid and was seen in the capillaries of the choroid, venules, and veins crossing through the interendothelial spaces [62]. In the dog, infusion of Evans blue dye into the CSF of the cisterna magna reveals drainage into the orbit resulting in proptosis [54]. Presumably this would occur through the aqueous humor-canal of Schlemn and nasolacrimal duct [63].

The most convincing evidence defining the anatomical basis for the CSF nasal lymphatic outflow system was demonstrated by Johnston *et al *that followed the outflow path of colored microfil injected into the CSF [64]. These anatomical preparations were carried out on pigs, rabbits, rats, mice, monkeys and seven days after death in humans. The lymphatic CSF drainage system was identified following the injection of yellow microfil into the CSF compartment. The microfil filled the subarachnoid space and entered the network of lymphatic vessels in the nasal submucosa in the animals studied. It generally appeared that in lower animals, the microfil did not enter the interstitium of the nasal mucosa but entered the lymphatic vessels directly from the perineural subarachnoid space. The actual configuration of this interface was not clear but the most likely arrangement is a direct connection with the lymphatic vessels since no microfil was found in the interstitium [64]. In humans, there were some extravasations into the interstitial space of the nasal mucosa that could have been primarily due to deterioration of the *post mortem *specimens. This study firmly established the anatomical basis for the lymphatic outflow system, which had been previously, demonstrated by the early semi-quantitative studies [3,48,51] and the volumetric studies which followed [44]. The actual microscopic configuration of the interface between the perineural space and the wall or lumen of the lymphatic vessels remains unknown. At physiologic pressure levels the injected microfil failed to demonstrate entry into the cranial venous system by way of the AV although this was not the case when the CSF pressure was elevated.

### Volumetric analysis of CSF lymphatic drainage system

Kida *et al *[65] confirmed from their studies in the rat that following the injection of India ink into the cisterna magna this tracer is primarily removed by way of the lymphatic system and less so by AV. This conclusion was supported by anatomical and not physiological evidence. This continuing view was generally supported in humans by *post mortem *examinations in fatal cases of subarachnoid hemorrhage where red cells have been found in both the nasal lymphatic system and the AV. Bradbury et al found that the percentage of the dose of tracer injected into the CSF found in the deep cervical lymphatics represented almost 30% of the CSF produced in the rabbit and 10-15% in the cat [3,57]. Johnston and his colleagues have investigated the functional capabilities of the lymphatic system in a series of distinctive publications based on experiments devised to provide quantitative evaluation of the various components of the total CSF drainage system in the sheep and rat. The volumetric studies were carried out primarily in conscious sheep in which one species of radiolabeled iodine was used to measure CSF outflow via the nasal lymphatic pathway and the cranial and spinal outflow system [66,67]. The second species of the radiolabeled iodine was used to correct for filtration of the CSF marker reaching the lymphatics from blood back into plasma from non-lymphatic drainage sites. It was assumed in the derived equations that the AV and lymphatic route were the only sites of absorption from the CSF system. It was reasonable to assume no significant loss of tracer occurred via the blood-brain barrier or choroid plexus. The mathematical model was based on a simple but reasonable three-compartment model (CSF, plasma, and lymph) and expected variations between animals both to isotope concentrations in various compartments and constancy or reliability of the anatomical arrangements. In the conscious animals, used in these experiments, it was expected that the pressures within the CSF compartments and the rate of fluid production and blood flow remained within physiological limits. They confirmed the conventional view that a portion of the CSF enters the superficial and deep cervical lymph nodes. Finding tracer in the lumbar and intercostal nodes indicated some spinal drainage of CSF [66,67]. The recovery of the radiolabeled human serum albumin (HSA) in both the lymphatic and AV drainage systems was accomplished by sampling from both the cannulated cervical lymphatic vessels and thoracic duct as well from venous blood. At 6 hours post injection into the cerebral ventricle 8.2% and 12.5% of the injected dose was recovered from the lymphatic and AV outflow respectively. At 22 hours the recovery was 25.1% and 20.8%. These findings demonstrated that clearance of the tracer was almost equally distributed between the lymphatic and AV drainage systems in their animal model [66]. There was no significant difference in the amount recovered in either outflow systems when the tracer was injected into the lumbar CSF. In a later study, the total rate of CSF absorption (lymphatic and AV routes) was estimated to be 3.48 ml/h [67].

Boulton *et al *studied the effect of pressure on the clearance of tracer from the ventricular system. They used a similar experimental arrangement in which the collection of the radiolabeled tracer from the lymphatic and venous systems was evaluated during a 3-hour low-pressure perfusion and a similar period for evaluating outflow at various pressure levels [68]. The results are shown in Table 2. On average an increase in ICP of 10 cm H_**2**_O elevated AV and lymphatic CSF clearance 2.7 and 3.9 fold respectively.

**Table 2:** Effect of pressure on cerebrospinal fluid outflow from ventricular system

**Pressure**^**1**^	**Lymphatic **^**2**^	**Arachnoid villi**^**2**^
0	0	0

10	0.1 - 0.56	0.88 - 1.52

20	0.12 - 3.8	2.3 - 6.44

30	0.04 - 18	0.68 - 12.04

^1 ^cmH_2_O ^2 ^ml/hr

Table modified and reproduced with permission from Boulton *et al *[68].

### The effect of blockage of cervical lymphatics and spinal subarachnoid space on drainage of CSF

When the cervical lymphatic vessels and cervical nodes were ligated significant intracranial consequences were reported such as elevation of ICP, EEG and behavioral modifications in the test animals [44]. These earlier studies suggested the importance of the lymphatics in maintaining a normal intracranial fluid homeostasis. The clearance of tracers from the CSF via the nasal lymphatic system following obstruction of the cribiform plate in rat revealed the recovered dose to be 0.697% before lymphatic ligation and 0.357% after ligation. It was concluded that ca. 50% of the outflow was by way of the lymphatics and the remainder due to both cranial and spinal AV drainage [69].

In order to evaluate the role of non-nasal lymphatic drainage of CSF via the cribiform plate in sheep, the cranial subarachnoid space was separated from the nasal outflow pathways by obstructing the cribiform plate and determining the impact on outflow resistance to CSF drainage. The CSF outflow resistance increased some 2.7 times and there was an associated elevation in ICP and increase in the time required for the pressure to fall to baseline after a bolus injection [70]. In neonatal sheep, Papaiconomou *et al *found that sealing the cribiform plate shifted the ICP - CSF flow (absorption) curve to the left [14]. When the cranial compartment was separated from the spinal compartment this effect on pressure-flow was enhanced. That is, that higher CSF pressures were required to handle CSF absorption. Transport of the radiolabeled tracer into cranial venous blood was also significantly increased under these conditions. This study would appear to indicate an important but secondary role of the cranial AV in CSF drainage, especially in the neonatal sheep with elevation of ICP, after isolating the lymphatic and spinal outflow systems. This secondary role might be expected since there is a relative paucity of villous structures along the superior sagittal sinus in this age group [71,72].

### Development and functional capabilities of the lymphatic CSF drainage system

The development of CSF absorption sites in rat and pig were studied in various dates before and following birth by Koh *et al *[73]. The CSF-lymphatic connections were evaluated utilizing colored microfil and a soluble Evan's blue protein complex. The pig was chosen because CSF synthesis occurs before birth while in the rat where CSF formation is markedly up regulated within a short time (a few weeks) following birth. In pigs, the CSF-Lymphatic connection were not observed at E80-81 fetuses but were observed as early as E92. In the rat, these connections were not seen until about a week after birth. It would appear that the onset of CSF formation correlated well with the establishment of the lymphatic outflow system in both species.

A comparison was made of CSF drainage between fetal, neonatal and adult sheep [14,74]. A summary of the outflow resistance and conductance is shown in table 3. The values in all age groups are quite similar with the highest concentration of the radiolabeled HSA tracer found in the cervical lymphatics. In rats and mice, the outflow resistance to CSF drainage increases at birth and then decreases steadily with age [75,76]. It was suggested that this was due to further development of the AV and correlated with the increase in CSF formation. It was also observed that a positive CSF-sagittal sinus pressure gradient was not observed which suggests a primary role of the lymphatics at this early time period. The global CSF transport rate and outflow resistance in neonatal period of the sheep was shown to be similar to that of the adult sheep (Table 3). This study [14] as with the earlier [74] study, that evaluated the fetal animal, indicates that even with fewer AV present early in life, there is a CSF outflow system with a capacity similar to the adult. The change in ICP and flow rate observed in the neonatal after isolating the cranial CSF compartment from the lymphatic olfactory outflow suggested to these authors that the AV play a limited role in early development and a secondary but more prominent role in the adult animal [44,73,74].

**Table 3:** CSF dynamics in fetal, neonatal, and adult sheep

	**R**_**out**_^**a**^	**Conductance**^**b**^	**Baseline ICP**^**c**^	**R**_**csf**_^**d**^
Fetal Sheep^1^	83.7 ± 9.8	0.013 ± 0.002	10.4 ± 0.8	6.3 ± 0.6

Neonatal Sheep^2^	96.5 ± 17.8	0.012 ± 0.003	6.6 ± 1.1	4.1 ± 1.2

Adult Sheep	84.7 ± 19.7	0.014 ± 0.003	11.9 ± 1.4	7.8 ± 0.7

^1 ^106-119 days gestation, ^2 ^2-6 days post birth, ^a ^cmH_2_O ml^-1^-min, ^b ^ml^-1^-min-cmH_2_O, ^c ^cmH_2_O ^d ^ml/hr. Data modified and reproduced with permission from Mollanji *et al *[74] and Papaiconomou *et al *[14].

The impact of aging on the lymphatic CSF in the rat was studied in Fisher 344 rats at age intervals from 3 to 19 months of age [77]. At 30 minutes post injection of ^125 ^I- HSA the % injected/g tissue from the nasal turbinates was 6.68 ± 0.42 at 3 months, 4.78 ± 0.67 at 6 months, 2.49 ± 0.31 at 12 months and 2.42 ± 0.72 at 19 months. This represented a significant decline in the drainage capacity of the CSF lymphatic system. The associated decrease in CSF formation with age also contributes to the decrease in CSF turnover found in the elderly [15].

### Hydrocephalus and impairment of CSF absorption by the lymphatic system

In rats in with kaolin induced communicating hydrocephalus, radio labeled HSA was injected into the lateral ventricle and the enrichment of the olfactory turbinates measured [78]. The presence of the ^125 ^I label in the turbinates was 0.99 ± 0.39 (% of injected/g tissue) in the hydrocephalic animals as compared to 5.86 ± 0.32 in the controls. It was suggested that the impairment of the subarachnoid pathway to and about the cribiform plate caused or contributed to the development of the hydrocephalus. This was followed up in a study by Rammling *et al *who studied the penetration of Evan's blue dye immediately following *post mortem *injection into the cisterna magna of congenital hydrocephalic H-Tx rats [79].The dye visualization in the olfactory region of the control Sprague-Dawley rats and the affected and unaffected H-Tx rats revealed less dye in the olfactory region of the hydrocephalic H-Tx rats. The conclusion, as with the kaolin-induced hydrocephalus, was that the hydrocephalus observed was primarily due to obstruction at the olfactory portion of the lymphatic CSF drainage system. It should be noted that there is a paucity of AV in rats, which suggests the primary role of the lymphatic outflow system in this species.

### Other sites for CSF absorption

The other possible sites for CSF absorption are the arachnoid membrane, choroid plexus, and the cerebral capillary. Utilizing the *ex vivo *model containing AV and adjacent arachnoid membrane. Grzybowski *et al*, using an *in vitro *AV preparation as an occluding membrane in a perfusion system demonstrated flow properties similar to that observed in an *ex vivo *AV perfusion preparation [24,25]. This suggested that the entire arachnoid membrane was capable of bulk CSF drainage [24]. This has yet to be demonstrated in the intact animal. Under normal circumstances it is possible that the Aquaporin4 found at the glial-endothelial cell interface could influence interstitial fluid absorption into the blood at physiological pressures although it has been convincingly demonstrated that the absorption and turnover of the cerebral interstitial fluid is adequately handled by the nasal lymphatic system [51]. The glucose and protein co-transporters GLUT1, KCC1, and MCT1 are found on the luminal and cerebral facing membranes of the endothelial cell. Although the total passive osmotic water permeability provided by these transporters maybe significant in brain homeostasis, their role in net water transport across the BBB is still not well understood [80]. The low permeability of the basal membrane and intercellular junctions of the choroid plexus as well as inadequate hydrostatic or osmotic gradients makes it unlikely that bulk movement of CSF into choroid plexus capillaries occurs under normal physiological conditions.

### CSF drainage in hydrocephalus

It has been proposed by Johanson *et al *that aquaporin1 (AQP1) and aquaporin4 (AQP4), at the blood-CSF and blood-brain barrier (endothelial cell of brain capillary) respectively, may be altered in experimental hydrocephalus [15]. Some compensation of the hydrocephalic situation would occur by the up-regulation of AQP4 at the glial-endothelial interface leading to enhancing the transport of cerebral interstitial fluid into blood while down-regulation of AQP1 at the choroid cell would diminish CSF formation. Both would tend to ameliorate the fluid accumulation in hydrocephalus. In communicating inflammatory hydrocephalus in rats it has been shown that periventricular AQP4 upgrade was strongly correlated with ventricular size. The AQP4 was first localized to the end feet of the astrocytes but later, the whole membrane of astrocytes became hypertrophic in severe cases of hydrocephalus [81]. A similar upgrade of this water channel protein has been reported in the H-Tx rat with spontaneously arrested hydrocephalus, which suggests a means of compensation for disturbed CSF absorption [82]. The effectiveness of changes in aquaporins in significantly ameliorating the hydrocephalic condition is not known but it suggests a protective response.

It has been shown that obstruction of one of the sites for CSF drainage leads to utilization of the remaining alternate sites. The drainage of CSF in both the AV (cranial and spinal) and lymphatic outflow systems increases dramatically with increasing CSF pressure [44,68]. It would therefore follow that this would occur in the intact system after one system outflow is diminished with elevation of outflow resistance and ICP. This is difficult to demonstrate since it is difficult to show that a pathological process that affects one pathway leaves the alternate pathways both morphologically and/or functionally intact. This is true of most inflammatory models (e.g., kaolin, hemorrhage, or infection) and certain congenital models. Even though subarachnoid hemorrhage in humans can lead to hydrocephalus with significant fibrotic changes in the AV, there can be changes in the patency of the subarachnoid space and evidence of red cells accumulation in the olfactory perineural pathways. Elevation of TGF-Beta 1 has been noted to result in hydrocephalus after subarachnoid hemorrhage induced in mice by intraventricular injection. It appeared that the main pathological change was interference in subarachnoid flow of CSF due to increased cellularity and fibrotic changes in the leptomeninx. It is difficult to separate the altered function of one site from others involved in CSF outflow under these conditions. In most experimental animals the time frame for maturation of the various outflow systems may also confound interpretation of capacity of alternate pathways in the very young animal [14]. The finding of an absence or paucity of arachnoid granulations in humans with hydrocephalus suggests the importance of these structures in total CSF drainage but the *post mortem *examination in these subjects had incomplete evaluation of the lymphatic system, global subarachnoid space and the spinal AV [30]. Following cranial base surgery, 8% of patients develop hydrocephalus in the postoperative period [83]. In this patient group, an even larger number developed CSF leaks which required shunting. It again is difficult to isolate the lesion site responsible. The observation that hydrocephalus is not usually seen after removal of anterior fossa tumors or following repair of CSF leaks due to interruption of olfactory rootlets overlying the cribiform area may support the importance of the cranial AV in CSF drainage in primates and a lesser role of the lymphatic drainage system in this species. In case of poor development of the olfactory bulbs it has been postulated that the potential loss of olfactory neurons might lead to loss of the conduit to the cervical lymphatics and thus leading to the observed hydrocephalus [44].

The importance of the spinal AV in CSF drainage seem supported by the observation that disturbing the cranial absorption of CSF following occlusion of the cisterna magna with kaolin resulted initially in a more than twofold increase in ICP and outflow resistance (R_o_). The ICP, but not R_o_, fell to normal values after six weeks [84]. The histological evaluation showed syrinx formation in the cervical and thoracic cord while marker proteins left the spinal subarachnoid space by way of the thoracic and lumbo-sacral rootlets. The presence of a continued rise of R_o _with a normal ICP at six weeks indicated a recruitment of spinal perineural outflow pathways for the compensation of disturbed cranial-CSF absorption [84]. In the rat, using a similar method of obstructing the cranial absorptive pathways, Voelz *et al*, using a ferritin tracer demonstrated passage from the central canal syrinx through ruptured ependymal and dorsal columns into extradural lymphatic vessels [85]. It was suggested that in the upright human the hydrostatic pressure differs significantly from the pressure in animals and therefore the spinal compensatory CSF outflow pathways may even be of greater importance. It should be noted that while patency rates of the central canal in humans is 100% under 1 year of age it markedly decreases beginning in the second decade of life. The occlusion of the central canal started at the T6 and L5 to S2 levels [86]. This potential pathway may not be operative after the second decade of life.

The question of alternative pathways after one of the CSF drainage site is anatomically altered maybe seen in hypovitaminosis A. It was noted in some studies that both a deficiency and excess of vitamin A could result in the animal developing hydrocephalus [31,87]. In these studies, there was no evaluation of the function of the potential alternate lymphatic route of absorption that may well have been compromised in these same animals. Hypo- and hypervitaminosis A does result in significant morphological changes in the AV. It has been reported that changes in the dura involve the mucopolysaccharides moiety and that since the AV are rich in mucopolysaccharides it could directly affect the integrity of these structures and decreased functional abilities [34,87,88]. Thinning of the AV cell cap and interstitial fibrosis have also been reported and associated with increased resistance to CSF outflow and absorption [88,89]. The fact that the observed increase in CSF pressure does not result in the development of hydrocephalus suggests that the lymphatic system may remain intact and provide an effective alternate pathway for CSF drainage.

### Dynamics of total CSF drainage

**Figure 12:**
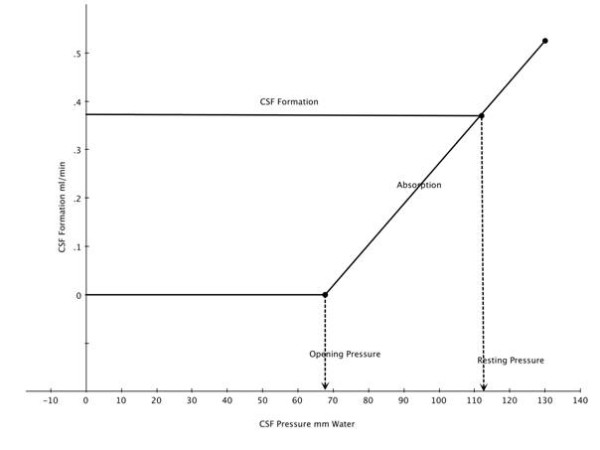
**Superimposed regression lines for CSF formation and absorption as a function of outflow pressure**. The intercept at 112 mm indicates the pressure at which formation and absorption are equal. The pressure at which absorption is zero is also indicated (modified and reproduced with permission from Cutler *et al *[90]).

In order to evaluate the relationship between CSF formation and absorption, Davson devised a variable infusion system that allowed control of infusion rate and pressure [3]. In the rabbit, the resistance to CSF outflow was more than 10 times greater than that found in the human. This difference correlates with the corresponding rate of CSF formation which in the rabbit is 10-12 μl/min while in human it is ca. 350 μl/min. He concluded that if the CSF pressures in all species are about the same that the resistance to outflow is inversely related to the volume of fluid flowing through the subarachnoid space. Cutler *et al*, [90] performed studies in children using a ventriculo-cisternal perfusion system in order to measure the relationship between CSF formation and absorption using a technique similar to that described by Davson and others [3]. The calculation of CSF formation and absorption in humans was based on the dilution of radio-labeled HSA in transit through the ventricular-spinal CSF pathways. Figure 12 demonstrates the curve-derived from this study The CSF absorption or outflow (ml/min) is zero until the outflow pressure (CSF pressure) exceeds 68 mm CSF. This is qualitatively similar to that observed in the perfusion of the isolated AV or recent studies of *in vivo *and *in vitro *fluid flow measured in a chamber with an AV membrane [5,24,25]. The intercept of the absorption and formation line crosses at a flow of ca 0.37 ml/min and a pressure of 112 mm CSF. The intercept represents equality between CSF formation and absorption. The pressure at the intercept indicates the normal horizontal resting pressure in these individuals. CSF absorption increases in a linear fashion with increasing CSF outflow pressure within the 68 - 250 mm CSF pressure that also has been noted in other constant infusion studies [90]. This study also revealed that CSF formation remains unchanged over this same range of pressures that has also been observed in human hydrocephalus [3]. Over the range of CSF pressures observed in this study the venous pressure in the superior sagittal sinus remained constant in relation to the CSF pressure. In hydrocephalic children, Shulman and Ransohoff [91] observed the usual differential pressure gradient in favor of CSF to sagittal sinus flow to be negated. In this same pressure range, R_0 _has been found to be pressure resistant but may deviate at considerably higher pressures [92]. Mann *et al *[93] using an intraventricular infusion system demonstrated that when the rate of infusion was at or below 1.0 ml/min the observed elevation of the CSF pressure achieved a steady state at that rate of infusion level. This implies that the CSF drainage or outflow system was still operative. When the infusion rate was increased above ca. 1.0 ml/min the pressure elevation that followed never reached a steady state. Under these conditions, the compliance of the venous system (volume reserve) was exhausted and the outflow system became inoperative. Presumably the CSF pressure at this rate of infusion led to collapse of the cranial venous system and loss of the CSF-venous pressure differential and cessation of absorption. The pressure at this rate of infusion reached > 900 mm H_2_0. A useful method for evaluating CSF compliance, absorption and formation is the bolus injection method [41]. This alternative method expresses the pressure-volume relationship as a pressure volume index (PVI), which is the volume, required to raise CSF pressure 10 fold. Compliance (C) can be computed using a single bolus injection and recording the volume injected (V) and the pressure at baseline (P_**o**_) and at the peak (P_**p**_) as V/log_**10 **_P_**p**_/P_**o**_. This bolus method not only allows computation of compliance (and the inverse: elastance) of the CSF system but also the outflow resistance of the absorptive pathways [41]. It was determined in the cat that about 2/3 of the compliance was in the cranial compartment (48% supratentorial and 20% infratentorial) and 1/3 in the spinal compartment [41]. The cranial compartment accounted for about 84% of total CSF absorption under normal conditions. The spinal absorptive capacity of 16% of total absorption suggests relatively minor role for the spinal arachnoid to compensate for challenges to volume accumulation within the CSF space [42]. Lofgren and Swetnow demonstrated a dramatic shift of the volume of distribution between the cranial and spinal compartments as the volume of CSF increased above the normal value leading to a reversal of the distribution of the volume accretion in favor of the spinal compartment [94]. The analysis of the Katzman and Hussey [95] studies in man by Marmarou *et al *[41] revealed the total PVI in man to be ca. 25 ml. and the distribution of compliance between cerebral and spinal compartments at 2 to 1.

**Figure 13:**
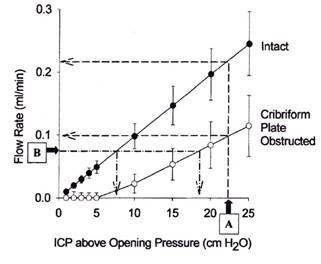
**Relationship between intracranial pressure (ICP) and flow rate (CSF absorption)**. CSF access to the spinal subarachnoid compartment was prevented. Closed circles represent data obtained before and open circles represent data obtained after the cribiform plate had been sealed. Opening pressure was the estimated threshold pressure at which CSF absorption was induced (reproduced with permission from Johnston and Papaiconomou [72]).

**Figure 14:**
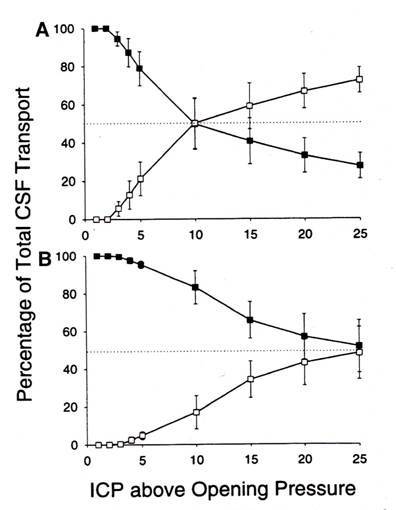
**Estimates of the proportion of total CSF transport through the cribiform plate (solid circles) and other pathways (cranial and spinal arachnoid villi) open circles in constant flow (A) and constant pressure (B) experiments**. Cribiform drainage is the dominant location at low and moderate ICP pressures. In A the spinal compartment is intact while in B the spinal compartment is blocked (reproduced with permission from Mollanji *et al *[100]).

Ekstedt [96] found the relationship between CSF outflow and pressure to be rectilinear up to a CSF pressure of ca. 6 kPa (61 cm H_2_0) after reaching a CSF pressure of ca. 60 mm H_2_0 required to initiate CSF outflow. This suggested to him that the AV when once opened are not further distended by pressure. It is apparent now that the outflow resistance and conductance that he measured in humans represents both the outflow via the cranial and spinal AV and the perineural lymphatic system. This suggests that the values for the total system outflow resistance (R_**o**_) and absorptive capacity path may represent a combined average value for both pathways. Using the bolus injection technique Sokolowski [97] constructed a series of pressure -time (PT) curves from which both a Pressure-volume (PV) and volume-time (VT) plot maybe derived. The PV plot represents the degree of distensibility of the system and the availability of the reserve space. The greater the pressure, the faster the excess fluid is drained from the system. The computed volume-time curves (VT) show the rate of absorption to be a function of pressure. The slope of this type of curve is assumed to be primarily determined by the drainage mechanism although the degree of elasticity in the compliance system is also a factor. The observed monoexponential curve represents the normal system where the CSF absorption is rapid and complete. When the absorptive mechanism(s) break down (as in the hydrocephalic patient) a bi-compartmental curve is noted and may indicate a defective, prolonged and incomplete absorption. This suggested to Sokolowski that global CSF absorption is composed of two outflow mechanisms; one a high and the other a low-pressure absorption system which work in harmony under normal conditions [97]. Alternatively this may also represent a secondary system for absorption in the presence of a primary absorption defect. Nelson and Goodman supported this concept and proposed that the CSF system was comprised of a series of valvular mechanisms with progressively higher opening threshold and either the lower or higher elements being selectively blocked [98]. This is in general agreement with Lorenzo *et al *[99] who studied the relationship between absorption of CSF and outflow pressure in human hydrocephalus. They observed type 1 absorption defect consisted of an opening pressure > 68 mmH_2_O but a rate of fluid absorption of 0.0069 ml/min/mm which was similar to the control value of 0.0076 ml/min/mm. The type 2 defect had a normal opening pressure but the rate of absorption was well below control at 0.0026 ml/min/mm. Another approach mentioned earlier is the sheep model where the outflow characteristics of the lymphatic and AV outflow systems were evaluated separately either by individual outflow collection or isolation due to physical obstruction [68-70,100]. The isolated collection methods indicated that both systems accounted for around half the CSF absorption. On average, an ICP increase of 10 cmH_2_O elevated AV and lymphatic CSF clearance 2.7- and 3.9-fold respectively. Earlier studies in the mature sheep [66,67] reveal the drainage of CSF via the lymphatic system to be 40-48% of the total. In young animals, the lymphatic outflow system appears to be dominant in large measure by the somewhat delayed development of the AV both in number and maturity [14,79]. Blocking of the olfactory perineural pathway at the cribiform plate impaired CSF transport significantly demonstrating the importance of the lymphatic system in total CSF drainage. In figure 13 it is clear that CSF absorption is significantly reduced after blocking CSF outflow via the cribiform plate in an animal in which access to the spinal compartment was denied [72]. In this experimental preparation, the outflow rate - ICP curve shifted to the right and the increase in outflow resistance required a greater pressure to achieve the same flow observed before obstruction. The ICP values are those above opening pressure. This study suggests that the cranial AV represent an outflow system operating at high CSF pressures as compared to that flow via the cribiform plate into the nasal lymphatic outflow system. Mollanji *et al *[100] blocked the cribiform plate and in some experiments also isolated the spinal compartment in sheep and challenged the system with constant flow or constant pressure infusions. The percentage of total CSF transport at various pressures above the opening pressure is presented in figure 14. It is assumed, that in the sheep, the opening pressure (OP) for CSF flow is similar to that observed in man (personal communication-Miles Johnston) and this seems to be the case in this study. From figure 14A and 14B, outflow begins to occur at about the same time above the opening pressure although the pressure required for non-lymphatic flow (cranial and spinal villi) to equal 50% of the total CSF transport is almost 10 cm above OP. When the spinal compartment is isolated by ligation (Figure 14B), the point at which the AV (cranial) and lymphatic systems reach 50% of total CSF transport is at a higher ICP and suggests the importance of the spinal AV system in the global absorption of CSF. This is not surprising since the spinal compartment has been shown to be responsible for up to ca. 25% of total CSF system drainage.

The maximum capacity of the CSF drainage systems to handle the volume of CSF produced before developing ventriculomegaly has not been fully defined. Measurement of CSF formation by external drainage has varied considerably in patients with a choroid plexus papilloma with associated hydrocephalus. In some cases the rate of formation determined in this manner were not much different than the normal range of values. However, there was no evaluation of the absorptive capacity of the outflow system measured at the same time of the drainage. This indicates that the outflow system might also be adversely affected in these patients]. The response of the CSF system to elevated rates of CSF formation (over production) has been studied in a patient with hydrocephalus associated with a choroid plexus papilloma by Eisenberg *et al *[101], using a ventriculo-lumbar perfusion system. They demonstrated that the absorption capacity of the total outflow system is exceeded at four times (1.43 ml/min) the rate of CSF formation in children (normal ~ 0.35 ml/min). The rate of absorption (V_a_) in this case was measured at 130 mmH_**2**_O and found to be 0.59 ml/min, which is almost equal to that found in normal children with unobstructed pathways (V_a _= 0.61 ml/min). Following the removal of the choroid plexus tumor, the intracranial pressure in the child returned to normal and the head size fell to the 50^th ^percentile for age. It can be concluded from this study that above the normal maximum absorptive level of ca 1.0 ml/min, the failure of brain compliance to accommodate the excess fluid and the effect of CSF pressure on the cranial venous system will result in accumulation of fluid and ventriculomegaly. This is in keeping with the perfusion studies by Mann in the dog which demonstrated the absorptive capacity of the CSF system is exceeded when the infusion into the supra cortical subarachnoid space was ≥ 1.0 ml/min [93].

## Conclusions

The evidence to date supports the importance of both the cranial arachnoid villi (AV) and lymphatic drainage systems in the egress of CSF from the subarachnoid space. The spinal AV have a lesser but important role especially when there is failure of the primary systems. The anatomy of both systems supports the view that both act as an open unidirectional pathway between the subarachnoid CSF and the vascular circulatory system. The lymphatic outflow system is primarily by way of the olfactory nerve perineural space that traverses the cribiform plate. Other cranial nerves have been implicated but there is little evidence to support an important role in CSF drainage. The cranial arachnoid system appears to allow CSF movement via both intracellular vacuoles and intercellular clefts. In primates they are primarily located along the dura of the superior sagittal sinus near the entrance of the parietal occipital vein, while in lower animals along the transverse sinus near the confluence of the sinuses. The lymphatic system has been shown to develop earlier than that of the AV and therefore appear to be a dominant CSF outflow route in the late fetal and early neonatal period. There is convincing evidence that the AV system loses it efficiency with age, which can influence the total turnover rate of the CSF with possible neurodegenerative consequences. The question of whether the obstruction of one of the systems can alone lead to hydrocephalus is still an open question as little convincing evidence is available that separates the status of the individual components in a clinical pathological setting

The global CSF drainage system in primates becomes operative at about 50 to 70 mm CSF pressure and normally flows out of the subarachnoid system at a differential pressure gradient of 3 to 4 mm Hg. The normal flow rate does vary with age but the average is ca. 0.35 ml/min. The maximum capacity of the global drainage system appears to be ca. 1.0 ml/min above which fluid accumulates in the ventricular system under increasing pressure. It is not possible to determine in primates the percentage of total CSF transport due to the lymphatic and AV outflow systems although it has been observed in humans that there is both a high and low pressure outflow system. It maybe reasonable to assume, based on the sheep studies, that the olfactory perineural lymphatic system is the lower pressure system. There is no experimental data to state with certainty the proportion of total CSF drainage due to either system at resting CSF pressure in primates.

## Competing interests

The authors declare that they have no competing interests.

## Authors' contributions

The sole author Michael Pollay is entirely responsible for the content of the manuscript.
